# Association between elder abuse and poor sleep: A cross-sectional study among rural older Malaysians

**DOI:** 10.1371/journal.pone.0180222

**Published:** 2017-07-07

**Authors:** Raudah Mohd Yunus, Syeda Wasfeea Wazid, Noran N. Hairi, Wan Yuen Choo, Farizah M. Hairi, Rajini Sooryanarayana, Sharifah N. Ahmad, Inayah A. Razak, Devi Peramalah, Suriyati A. Aziz, Zaiton L. Mohamad, Rosmala Mohamad, Zainudin M. Ali, Awang B. Awang Mahmud

**Affiliations:** 1 Julius Centre University of Malaya (JCUM), Department of Social and Preventive Medicine, University of Malaya, Kuala Lumpur; 2 Department of Population Health and Preventive Medicine, Universiti Teknologi MARA, Sungai Buloh, Selangor; 3 Negeri Sembilan State Health Department (JKNNS), Negeri Sembilan, Malaysia; Universidade Federal do Rio de Janeiro, BRAZIL

## Abstract

**Objectives:**

To examine the association between elder abuse and poor sleep using a Malay validated version of Pittsburgh Sleep Quality Index (PSQI).

**Design:**

This study was divided into two phases. Phase I tested the construct validity and reliability of the Malay version of PSQI. Phase II was a population-based, cross-sectional study with a multi-stage cluster sampling method. Home-based interviews were conducted by trained personnel using a structured questionnaire, to determine exposure and outcome.

**Setting:**

Kuala Pilah, a district in Negeri Sembilan which is one of the fourteen states in Malaysia.

**Participants:**

1648 community-dwelling older Malaysians.

**Results:**

The Malay version of PSQI had significant test re-test reliability with intra-class correlation coefficients of 0.62. Confirmatory factor analyses revealed that one factor PSQI scale with three components (subjective sleep quality, sleep latency, and sleep disturbances) was most suitable. Cronbach’s Alpha was 0.60 and composite reliability was 0.63. PSQI scores were highest among neglect (4.11), followed by physical (4.10), psychological (3.96) and financial abuse (3.60). There was a dose-response relationship between clustering of abuse and PSQI scores; 3.41, 3.50 and 3.84 for “no abuse”, “1 type of abuse” and “2 types or more”. Generalized linear models revealed six variables as significant determinants of sleep quality–abuse, co-morbidities, self-rated health, income, social support and gait speed. Among abuse subtypes, only neglect was significantly associated with poor sleep.

**Conclusion:**

The Malay PSQI was valid and reliable. Abuse was significantly associated with poor sleep. As sleep is essential for health and is a good predictor for mortality among older adults, management of abuse victims should entail sleep assessment. Interventions or treatment modalities which focus on improving sleep quality among abuse victims should be designed.

## Introduction

Elder abuse and neglect (EAN) is increasingly becoming a global health concern. The World Health Organization (WHO) has defined EAN as ‘a single or repeated act, or lack of appropriate action, occurring within any relationship where there is an expectation of trust which causes harm or distress to an older person’[1]. There are five subtypes of elder abuse–physical, psychological, financial, sexual and neglect [2]. Worldwide prevalence of EAN was estimated at 3.2% to 27.5% [3]. In Malaysia, one study reported a 12-month prevalence of 9.6% among urban elders [4] while another reported a lifetime prevalence of 8.1% among those in the rural area [5].

Numerous adverse effects of abuse in late life–across different health domains—have been documented. These include premature mortality [6, 7], depression [8], increased utilization of health services [9, 10], metabolic syndrome [11], musculoskeletal pain, suicidal ideation, anxiety, incontinence and gastro-intestinal symptoms [12]. Nevertheless, many health-related outcomes of EAN remain unexplored. A conceptual framework by Anetzberger on the health effects of elder abuse has categorized them as physical, psychological, behavioural and social [13].

Quality sleep is one of the key determinants of good health. Its importance is more prominent among older adults, as sleep disorders and sleeping difficulties become more pervasive with aging [14]. Poor sleep has been found not only to predict mortality [15, 16] and nursing home placement among older individuals [16], but it was also associated with obesity [17], cognitive decline [18] and diabetes [19]. Despite this, the relationship between abuse experience in late life and sleep quality has not been adequately explored. Olofsson et al reported that older Swedish victims of physical and psychological abuse were more likely to experience sleeping problems than those who were not abused [12]. Other than this, there has been no investigation to corroborate this finding and whether or not it is replicable across different populations remains a question. The issue of cross-cultural differences in the manifestations of EAN outcomes has been raised [20]. Culture is said to influence the expression of bodily symptoms [21], sleep habits and subjective appraisal of sleep [22]. As argued by Stranges et al, “Sleep habits are multifaceted and result from a complex interplay between genetics, environment, and social factors, as well as the presence of comorbidities. Factors contributing to sleep problems in older adults from low income countries may differ from those characteristic of Western societies”[22].

Sleep can be measured either objectively or subjectively. Objective measurement of sleep often employs polysomnography (PSG) and the Multiple Sleep Latency Test (MSLT), but these methods have been considered impractical for clinical screening and research [23]. The more common approach in research therefore has been the subjective measurement using questionnaires–the most widely used being the Pittsburgh Sleep Quality Index (PSQI) and Epworth Sleepiness Scale (ESS) [23]. In this study, we have employed the PSQI for its compatibility with our study objectives, that is to measure the overall sleep quality among those abused and not abused, and discriminate between ‘good’ and ‘poor’ sleepers [24, 25]. The ESS on the other hand has been designed more specifically for assessing daytime sleepiness [25, 26].

The PSQI is a nineteen-item questionnaire measuring seven components of sleep–subjective sleep quality, sleep latency, sleep duration, habitual sleep efficiency, sleep disturbances, use of sleeping medication, and daytime dysfunction–yielding one global score [24]. Most studies posit a single factor structure of PSQI comprising the seven components scores. However, some studies have posited a two-factor model and a three-factor model [27, 28]. The two-factor model consists of sub-scales sleep efficiency (components three and four), and perceived sleep quality (the remaining components). The three-factor model consists of sub-scales sleep efficiency (components three and four), perceived sleep quality (components one, two and six), and daily disturbances (components five and seven). PSQI has been translated into various languages and is widely used in research studies [29]. Cross-cultural validation has also been conducted among Brazilian, Israeli, Nigerian, Italian, Korean and Arab samples [29–33]. The Malay version of PSQI was used in a number of studies [34–37], but validation was never attempted. In the current study all these models were explored to identify the most suitable factor structure for the Malaysian context.

We thus aim to: 1) validate the Malay version of PSQI among older Malaysians through confirmatory factor analyses (CFA) and reliability tests, and; 2) examine the association between EAN and sleep quality.

## Methods

This study consisted of two phases. In Phase 1, we validated the Malay version of PSQI and in Phase 2, we examined the relationship between EAN and sleep quality. Assessment was carried out in a span of six months, from November 2013 to May 2014.

Phase 1: Validation of Malay PSQI.

Eight low-cost, subsidized government flats were randomly chosen from a list of all low-cost flats in the Klang Valley. A total of 239 residents aged 60 or more were then randomly selected and invited to join the study. The Malay version of PSQI was administered face-to-face by trained personnel two times, in a two-week interval.

The PSQI is a self-rated questionnaire comprising nineteen questions with an additional five questions rated by another person such as bed-partners or roommates. The five questions were not administered since they are usually used for clinical purposes and not included in the scoring system. According to the guidelines of Buysse et al. [24] the nineteen items were transformed into seven components: subjective sleep quality, sleep latency, sleep duration, habitual sleep efficiency, sleep disturbance, use of sleeping medication, and daytime dysfunction. Each component score ranged from 0 to 3, where 0 indicated “no difficulty” and 3 indicated “severe difficulty”. All components were then summed to yield a global PSQI scores ranging from 0 to 21. Higher scores indicated poorer sleep. In our study, the validated Malay version retained three components (consisting of twelve items) and therefore had a score range of 0 to 9.

Phase 2: EAN and sleep quality.

### Study design

This was a cross-sectional study, involving community-dwelling older Malaysians aged 60 or more. It was part of the Malaysian Elder Mistreatment Project (MAESTRO) initiated in early 2013 [38].

### Setting

The district of Kuala Pilah in Negeri Sembilan, a state in Peninsular Malaysia was chosen. Largely rural, Kuala Pilah is located roughly 100 km away from the capital city, Kuala Lumpur.

### Sampling strategy

A multi-stage cluster sampling strategy was employed. Kuala Pilah was first randomly selected from Negeri Sembilan’s seven districts. Out 254 enumeration blocks (EB) in the district, 156 EBs were randomly chosen, followed by another random selection of sixteen to twenty households from every EB based on a computer-generated list. A house-to-house visit was then performed to identify selected participants.

Our study respondents were restricted to those aged 60 and older, Malaysian nationals, had taken up residence in Kuala Pilah for at least 12 months, and those who were able to communicate independently. We excluded residents of long-term care institutions, those unable to communicate for reasons such as severe cognitive impairment, deafness or severe hearing impairment and post-stroke complications, and those who scored 4 or less in the Mini Mental State Examination (MMSE).

Respondents were interviewed at home by trained personnel using a structured questionnaire. A number of 1648 older respondents successfully completed the interviews. Further details on the study methodology have been previously published [38].

### Measures

#### Elder abuse and neglect (EAN)

Elder abuse and neglect (EAN) was captured using a questionnaire derived from the modified Conflict Tactic Scales (CTS) and revised by Naughton and colleagues [39]. Each respondent was asked a series of questions in which they gave a binary response of ‘yes’ or ‘no’, followed by the frequency of abusive episodes and how serious the event was. Abuse was categorized into five sub-types; physical, psychological, financial, sexual and neglect. A more detailed description of questionnaire items and how EAN was operationalized is available in the published study protocol [38].

#### Sleep quality

Sleep quality in this study was assessed using the Malay validated Pittsburgh Sleep Quality Index (PSQI) as described above. Higher scores indicate poorer sleep, as suggested by Buysse et al. [24]. Scores ranged from zero to nine. In this paper we used the terms ‘poor sleep’, ‘sleep difficulties’ and ‘sleep disturbance’ interchangeably.

#### Covariates

Other variables measured were: 1) socio-demographics (age, sex, ethnicity, household income and education level); 2) health-related variables (number of co-morbidities and self-rated health); 3) psychosocial variables (depression and social support) and; 4) physical function (2.4-meter gait speed).

Monthly household income was categorized into “low” (less than RM1000), “medium” (RM1000-RM2499) and “high” (RM2500 and above). Education level was grouped into “low” (no formal education), “medium” (primary to secondary) and “high” (college and above). Depression was determined using the Geriatric Depression Scale (GDS-15) [40], while social support was measured by the DUKE Social Support Index (DSSI) [41].

### Analytic approach

Data was analyzed with the IBM Statistical Package for Social Science (SPSS) software version 20.0 for Windows [42]. Validation was performed through confirmatory factor analysis (CFA) with the weighted least squares method with mean and variance adjustment estimator as the data was non-normal. Reliability was assessed in two ways: a) internal consistency reliability measured by Cronbach’s alpha and composite reliability, and; 2) test-retest reliability measured by intra-class correlation (ICC) coefficient. For Phase II, missing data was addressed by multiple imputation (Markov Chain Monte Carlo) method. Continuous data was reported in means and standard deviation (SD), whereas categorical data was reported in percentages. Comparison between two and three groups of continuous variables was done using the independent sample t-test and ANOVA, respectively. The Pearson’s Correlation Coefficient was employed to quantify associations between continuous variables, while Pearson’s Chi Squared test was used to determine relationships between categorical variables.

To study the relationships between independent and dependent variables, Generalized Linear Models (GLiM) were employed; using Maximum Likelihood Estimation (MLE) as the method of parameter estimation, distribution of independent variable (PSQI) set as Gamma (positive values with skewness to the right), and ‘Power’ as the link function. Multi-collinearity diagnostics was run prior to analyses, and interactions between EAN and other predictor variables (age, sex, income, education, co-morbidities, self-rated health, gait speed, depression and social support) were examined. Ethnicity was collapsed into two groups; “Malay” and “Non-Malay”, given the very small sample size of each of the non-Malay ethnic groups (Chinese, Indian and Aborigine). A *p*-value of less than 0.05 was considered statistically significant and 95% CIs were reported.

### Ethics

This study was approved by the Medical Ethics Committee of the University of Malaya Medical Centre (MEC Ref 902.2) and the Medical Research and Ethics Committee, Ministry of Health Malaysia (NMRR-12-1444-11726). Thorough explanation was given to each respondent and written consent was taken prior to interview.

## Results

Phase 1: Validation of Malay PSQI.

Out of 239 older adults invited, 194 agreed to participate in the validation study. Response rate was 81.2%. Those found to have severe depression (GDS scores 12 or more) and cognitive impairment (MMSE scores 17 or less) were excluded. The final sample size was 183. Descriptive statistics of all PSQI components are shown in Appendix 1 (S1 Appendix). CFA was then run on the original models (one factor, two factor, three factor) consisting of all the seven components. Appendix 2 (S2 Appendix) demonstrates CFA model fit statistics, item loadings, and reliability values for the different PSQI models.

**Fig 1:**
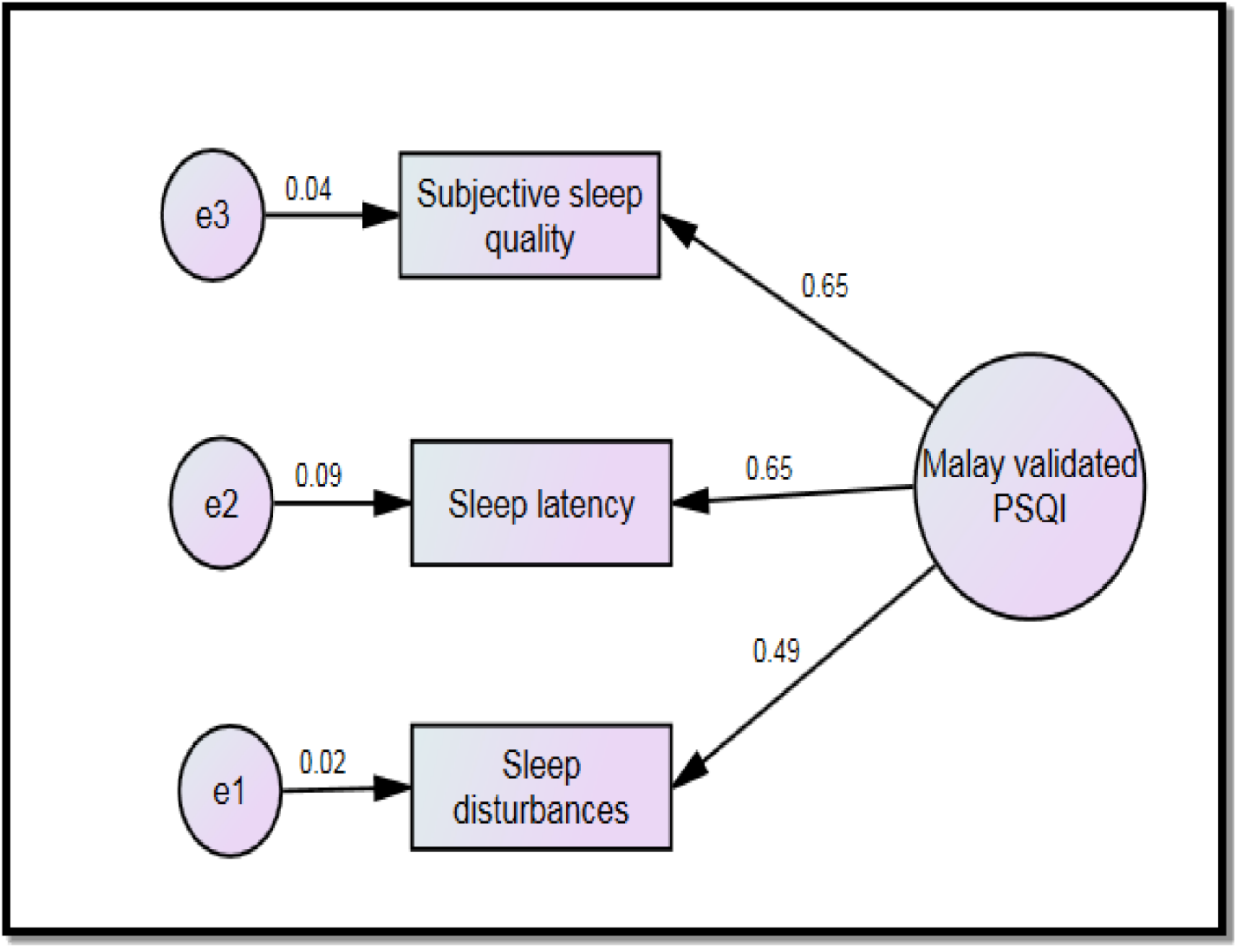
The final CFA model of the Malay validated PSQI.

The overall model fit indices for the original models (one factor, two factor, three factor) were acceptable but the item loadings were poor. Components 3,4,6 and 7 were then dropped one by one and finally PSQI with three components (subjective sleep quality, sleep latency and sleep disturbances) was found appropriate, demonstrating good convergent validity. The final scale had acceptable Cronbach’s alpha and composite reliability, and significant ICC of 0.61 (S3 Appendix). The final Malay validated PSQI model is presented in Fig 1.

Phase II: EAN and sleep quality.

A total of 2118 individuals agreed to participate. Response rate was 84.9%. There was no difference between respondents and non-respondents with regards to socio-demographic characteristics. Screening of cognitive impairment using MMSE excluded 191 individuals, and 279 did not show up on the day of PSQI interview (unit non-response), yielding a final sample size of 1648. Those who did not undergo PSQI interview were older, while other characteristics did not differ. The sample comprised of 97.4% Malay, 60.2% women, and slightly more than half belonged to the younger age group (60–69). Missing data was found to be MAR (missing at random), and this was addressed by multiple imputation method.

The prevalence of EAN overall was 8.1% (n = 133). Financial abuse was the most common subtype, comprising 4.9% while psychological, physical, neglect and sexual abuse were 3.3%, 1.2%, 1.2% and 0.2% respectively. Unadjusted analyses showed that abuse had significant relationships with low income, depression and poor sleep quality. Mean PSQI scores for EAN victims were 3.80 (SD = 1.55), whereas for those not abused, mean scores were 3.41 (SD = 1.38). Table 1 illustrates the basic characteristics of our study respondents.

**Table 1:** Basic characteristics of study respondents (N = 1648).

Variable	All N (%)	Abused N (%)	Not Abused N (%)	*p*
Age				
60–69	857(52.1)	66(49.6)	791(52.3)	0.73
70–79	642(39.0)	53(39.8)	589(38.9)	
80 or more	147(8.9)	14(10.5)	133(8.8)	
Sex				
Male	655(39.8)	59(44.4)	596(39.4)	0.26
Female	992(60.2)	74(55.6)	918 (60.6)	
Ethnicity				
Malay	1605(97.4)	59(44.4)	1476(97.5)	0.64
Chinese	13(0.8)	2(1.5)	11(0.7)	
Indian	21(1.3)	2(1.5)	19(1.3)	
Aborigine	8(0.5)	0(0)	8(0.5)	
Household income				
Low	1059(64.3)	99(74.4)	959(63.3)	0.02*
Medium	530(32.2)	29(21.8)	502(33.1)	
High	59(3.5)	5(3.8)	54(3.6)	
Education level				
Low	199(12.1)	11(8.3)	188(12.4)	0.34
Medium	1415 (85.9)	120 (90.2)	1295 (85.5)	
High	33(2.0)	2(1.5)	31(2.1)	
Self-rated health				
Poor	436(26.5)	44(33.1)	392(25.9)	0.07
Good	1209(73.5)	89(66.9)	1120(74.1)	
No. of co-morbidities				
Mean (SD)	1.47(1.30)	1.57(1.42)	1.46(1.33)	0.34
Depression				
Mean (SD)	3.94(3.70)	4.82(3.90)	3.88(3.7)	0.01*
Social support				
Mean (SD)	27.4(3.2)	26.9(3.9)	27.5(3.1)	0.17
Gait speed				
Mean (SD)	6.60(2.50)	6.49(1.90)	6.61(2.50)	0.64
Sleep quality				
Mean (SD)	3.44(1.40)	3.80(1.55)	3.41(1.38)	0.01*

*p<0.05

**Fig 2:**
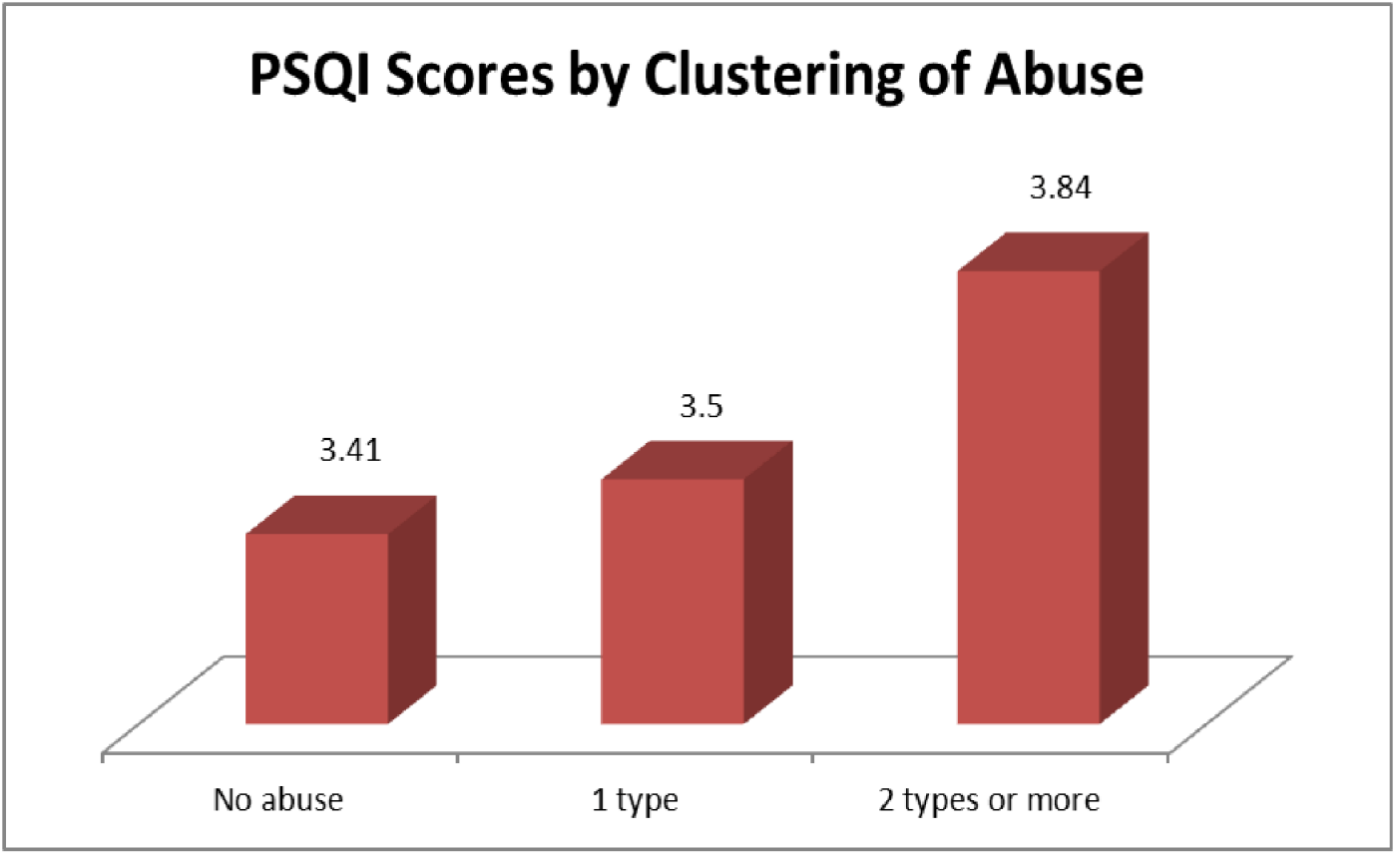
Dose-response relationship between number of abuse and PSQI scores.

Mean PSQI scores were highest in neglect (4.11, SD = 1.7), followed by physical (4.10, SD = 1.1), psychological (3.96, SD = 1.7), and financial abuse (3.60, SD = 1.4). Sexual abuse had to be excluded from analysis due to the extremely small number of cases (n = 5). A dose-response relationship was evident between clustering of abuse and PSQI scores; 3.41 (SD = 1.3), 3.50 (SD = 1.4) and 3.84 (SD = 1.3) for “no abuse”, “1 type of abuse” and “2 types or more”, respectively. Fig 2 demonstrates the graded relationship between clustering of abuse and PSQI scores.

Analyses using Generalized Linear Models were then performed to determine the relationship between independent variables and PSQI scores. There was no multi-collinearity between the any of the predictor variables. Six were found to be significantly associated with poor sleep (higher PSQI scores); 1) EAN–abuse victims were more likely to have poorer sleep than those not abused; 2) co-morbidities–those with multiple chronic diseases were more likely to have poor sleep than those with no or few diseases; 3) self-rated health; those who rated their health as “good” were more likely to have better sleep than those who rated their health as “poor”; 4) income; those with lower income had poorer sleep; 5) social support; those with lower social support were more likely to have higher PSQI scores, and; 6) gait speed; those with lower gait speed had poorer sleep. No interaction was found between abuse and other variables in affecting sleep quality. When similar analyses were run with overall EAN being replaced by the four subtypes–physical, psychological, financial and neglect–only neglect was associated significantly with decline in sleep quality.

Table 2 shows results of GLiM analyses to determine the associations between independent variables and PSQI scores, while Table 3 shows the associations between EAN subtypes and PSQI scores.

**Table 2:** Relationship between abuse, socio -demographics, physical function, health-related and psychosocial variables with PSQI scores.

Variables	B	SE	95% CI	*p-value*
Age	-0.001	0.001	-0.001, 0.001	0.190
Sex [male]	-0.002	0.006	-0.014, 0.011	0.801
Ethnicity [Non-Malay]	0.014	0.018	-0.02, 0.049	0.413
Income [low]	-0.038	0.019	-0.076, -0.010-	0.046*
Income [middle]	-0.045	0.019	0.083, -0.007	0.020*
Education [low]	-0.020	0.025	-0.070, 0.029-	0.421
Education [medium]	0.007	0.024	0.054, 0.039	0.765
No. of co-morbiditis	-0.010	0.002	-0.014, -0.006	**<0.001** **
Self-rated health [poor]	-0.024	0.007	-0.037, -0.011	**<0.001** **
Gait speed	-0.003	0.001	-0.005, 0.001	0.025*
Depression	-0.001	0.001	-0.002, 0.001	0.431
Social support	0.002	0.001	0.001, 0.004	0.030*
Abuse [yes]	-0.026	0.010	-0.045, -0.007	0.007**

*p<0.05

**p<0.01, Deviance: 276.38, Pearson’s Chi-Square: 254.42, AIC = 5587.4

**Table 3:** Relationship between EAN subtypes and PSQI scores.

Variable	B	SE	95% CI	*p-value*
Physical	0.04	0.024	0.09,0.01	0.09
Psychological	0.03	0.016	0.06,0.01	0.11
Financial	0.01	0.014	0.02,0.04	0.55
Neglect	0.05	0.023	0.10,0.01	0.03*

*p<0.05, Deviance: 250.59, Pearson’s Chi Square: 231.78, AIC = 5020.4

## Discussion

Our study showed that, overall, respondents with abuse experiences had significantly poorer sleep than those who were not abused. Unadjusted analyses showed associations between EAN and low income and depression (Table 1). This corroborated previous findings which reported low income and psychological or psychiatric problems as risk factors for elder abuse [43]. Besides, depression was also an established mental health outcome of EAN [44–46].

The graded relationship between clustering of abuse and PSQI scores strengthened the association between EAN and poor sleep–as abuse experience is multiplied, sleep quality declines further as demonstrated by the steady increase in PSQI scores. This can be explained by the link between stress–with EAN being a form of stressor–and sleep problems as reported by a number of studies: 1) high levels of emotional arousal as a result of adverse events were said to cause sleep difficulties [47]; 2) exposure to stress increased the risk of insomnia [48], and; 3) sleep recordings demonstrated that stress was associated with “shortened sleep, fragmentation, and possibly a reduction in sleep stages 3 and 4”, which then led to higher levels of cortisol, thus exacerbating stress [49]. Other forms of family violence–child abuse and intimate partner violence (IPV)–have all been shown to be associated with sleep disturbances [50, 51].

Among the subtypes of abuse, neglect had the strongest association with poor sleep. Similarly, mean PSQI scores were highest in neglect. The operational definition of neglect in this study included: 1) not being provided with support for basic activities of daily living (cooking, bathing, dressing, walking, climbing stairs, etc), and; 2) being deprived of safe and clean living conditions, medical attention or treatment, clean clothing, and adequate, nutritional food [38]. The dynamics between neglect and sleep problems may be explained by different pathways. First, a direct pathway–lack of access to proper shelter could indicate that the victim is made to live in a poor condition that prevented him from obtaining adequate, comfortable sleep. Second, an indirect pathway–deprivation of medical attention and nutritional food could result in various health problems or aggravate existing illnesses. As a consequence, sleep difficulties arise. Studies have shown that poor sleep among older individuals was associated with co-morbidities and health status [52, 53]. Similarly, limitations in activities of daily living–a component of neglect–was related to sleep disturbance [53]. Third, the overall effects of neglect can be greater than other individual subtypes of abuse, due to its multi-dimensional nature. Some components of neglect overlap with psychological and financial abuse; neglect victims inevitably suffer, in a subtle manner, from psychological distress and constraint resources. Fourth, neglect is chronic in nature–established by at least ten occurrences in our study [38]. Unlike other subtypes such as financial or physical abuse where a single episode of mistreatment was considered abuse, neglect victims tend to suffer longer, thus giving enough time for changes in sleep to manifest.

Other variables found to be significantly associated with sleep quality were self-rated health, co-morbidities, gait speed, income and social support. These findings have been substantiated by previous studies; older adults who rated their health positively, or had no or less co-morbidities reported better sleep in comparison with those with poor self-rated health and more chronic diseases [53, 54]. Low income had also been linked to poor sleep quality [55]. Similarly, physical function–represented by gait speed in this study–was associated with sleep difficulties. Dam et al reported a relationship between lower gait speed and greater sleep fragmentation, poorer sleep efficiency and sleep hypoxia among older American men [56], while Goldman et al found that older women with poor physical performance (including gait speed) had greater sleep disturbances [57]. Sleep quality among older adults was also said to be influenced by their social support and network [58, 59].

On the other hand, depression was not associated with poor sleep in our study, in contrast with prior findings which suggested a relationship between the two [60, 61]. However, a study on subjective sleep quality characteristics among older adults found that of all PSQI components, usage of sleeping medication was the only one associated with depression [62]. This component (use of sleeping medication) was among the four components dropped in our validated Malay PSQI. The relationship between depression and poor sleep in later life therefore may be more complex than the conventional assumption, thus warranting further investigation.

Given that our findings supported an association between EAN and poor sleep, current approach to managing abuse victims needs to be tailored accordingly. Healthcare providers should always be alerted to the possibility of sleeping problems when dealing with EAN victims, especially when there are elements of neglect. On top of that, detection of co-existing multiple chronic diseases, poor self-rated health and low social support–all of which can be easily identified–may be a simple yet effective technique to screen for sleeping problems among abuse victims, prior to confirmation with more sophisticated methods. Improvement of sleep quality therefore should be one of the aims while managing EAN victims. This can be done either through designation of specific treatment modalities by clinicians or a more general form of intervention by public health experts and policy-makers.

This study has several limitations. First, its cross-sectional design disallows causality. The possibility of the relationship between EAN and poor sleep in the opposite direction cannot be ruled out. Second, PSQI is a subjective measurement of sleep. Its accuracy may differ from objective methods such as polysomnography. It was reported that differences in results were found between the two methods, and that the correlation between self-reported and objectively-measured sleep could be biased [63, 64]. Third, our study excluded older adults with severe cognitive impairment, severe hearing impairment and inability to communicate independently. In addition, those who did not turn up for sleep assessment (through PSQI questionnaire) comprised the older group. This could have underestimated the prevalence of sleep disturbance and strength of association between abuse and sleep quality. Fourth, validation of the Malay PSQI was conducted among low-cost flat residents. This could have resulted in potential bias, as those from the higher income group were not included. However, the majority of our rural respondents in Phase II were classified as ‘low-income’ based on their monthly household earning, while the high-income group constituted only 3.5%. Nevertheless, our results need to be interpreted in the light of these constraints.

## Supporting information

S1 Appendix Descriptive statistics of all the components of the original PSQI. (DOCX)

S2 Appendix CFA model fit statistics, item loadings, and reliability values for the different PQSI models. (DOCX)

S3 Appendix Test-retest reliability of the PSQI scores assessed by Spearman’s and intra-class correlation coefficient. (DOCX)

S1 File Study data (data_EAN.sav). (SAV)
