# Correction: Association between elder abuse and poor sleep: A cross-sectional study among rural older Malaysians

**DOI:** 10.1371/journal.pone.0187782

**Published:** 2017-11-02

**Authors:** Raudah Mohd Yunus, Syeda Wasfeea Wazid, Noran N. Hairi, Wan Yuen Choo, Farizah M. Hairi, Rajini Sooryanarayana, Sharifah N. Ahmad, Inayah A. Razak, Devi Peramalah, Suriyati A. Aziz, Zaiton L. Mohamad, Rosmala Mohamad, Zainudin M. Ali, Awang Bulgiba

The last author’s name is incorrect. The correct name is: Awang Bulgiba.
