# Supplementary material for: Association between elder abuse and poor sleep: A cross-sectional study among rural older Malaysians
Source: PLoS One. 2017 Jul 7;12(7):e0180222. doi: 10.1371/journal.pone.0180222 (PMC5501458; doi:10.1371/journal.pone.0180222)
Supplement: S1 Appendix — (DOCX) [file pone.0180222.s001.docx]

Appendix 1

Descriptive statistics of all the components of the original PSQI

| Component | Mean | SD | Skewness | Kurtosis | Item-Total Correlation |
| --- | --- | --- | --- | --- | --- |
| 1. Subjective sleep quality | 0.90 | 0.59 | 0.20 | 0.58 | 0.33 |
| 1. Sleep latency | 0.92 | 0.93 | 0.75 | -0.32 | 0.26 |
| 1. Sleep duration | 1.11 | 0.98 | 0.21 | -1.23 | 0.01 |
| 1. Habitual sleep efficiency | 0.05 | 0.34 | 8.03 | 66.44 | 0.05 |
| 1. Sleep disturbances | 1.00 | 0.50 | 0.01 | 1.09 | 0.24 |
| 1. Use of sleeping medication | 0.02 | 0.19 | 8.75 | 82.19 | 0.17 |
| 1. Daytime dysfunction | 0.05 | 0.21 | 4.30 | 16.70 | 0.01 |

It was observed from table 1 that items 4, 6, had 7 had low Mean and SD, and was highly skewed and kurtotic. The data was therefore considered non-normal. Following this, CFA was run.
