# Supplementary material for: Association between elder abuse and poor sleep: A cross-sectional study among rural older Malaysians
Source: PLoS One. 2017 Jul 7;12(7):e0180222. doi: 10.1371/journal.pone.0180222 (PMC5501458; doi:10.1371/journal.pone.0180222)
Supplement: S2 Appendix — (DOCX) [file pone.0180222.s002.docx]

Appendix 2

CFA model fit statistics, item loadings, and reliability values for the different PQSI models

|  | One factor  model | Two-factor  model | Three-factor  model | One factor#  (final model) |
| --- | --- | --- | --- | --- |
| Model fit indices:   1. CFI 2. TLI 3. RMSEA 4. SRMR | 0.94 0.92 0.03 0.06 | 0.92 0.88 0.03 0.06 | 0.93 0.87 0.04 0.05 | 1.00 1.00 0.01 0.01 |
| Item loadings:   1. Subjective sleep quality | 0.67 | 0.67 | 0.67 | 0.65 |
| 1. Sleep latency | 0.64 | 0.64 | 0.64 | 0.65 |
| 1. Sleep duration | -0.14 | 0.25 | 0.25 | - |
| 1. Habitual sleep efficiency | 0.01 | 0.75 | 0.75 | - |
| 1. Sleep disturbances | 0.49 | 0.49 | 0.71 | 0.49 |
| 1. Use of sleeping medication | 0.01 | 0.01 | 0.01 | - |
| 1. Daytime dysfunction | 0.04 | 0.04 | 0.07 | - |
| Cronbach’s alpha of the factors: | 0.27 | - | - | 0.60 |
| 1. Sleep efficiency | -  -  - | 0.21 | 0.21 | -  -  - |
| 1. Perceived sleep quality |  | 0.50 | 0.43 |  |
| 1. Daily disturbances |  | - | 0.07 |  |
| Composite reliability of the factors: | 0.40 | - | - | 0.63 |
| 1. Sleep efficiency | -  -  - | 0.42 | 0.42 | -  -  - |
| 1. Perceived sleep quality |  | 0.47 | 0.45 |  |
| 1. Daily disturbances |  | - | 0.29 |  |

Note: n= 183; One factor^#^  is the final model with components 3,4,6, and 7 removed.
