# Supplementary material for: Association between elder abuse and poor sleep: A cross-sectional study among rural older Malaysians
Source: PLoS One. 2017 Jul 7;12(7):e0180222. doi: 10.1371/journal.pone.0180222 (PMC5501458; doi:10.1371/journal.pone.0180222)
Supplement: S3 Appendix — (DOCX) [file pone.0180222.s003.docx]

Appendix 3

Test-retest reliability of the PSQI scores assessed by Spearman’s and intra-class correlation coefficient

|  | **Mean ± SD** | **Mean ± SD** | **Spearman correlation coefficient** | **ICC** | **95% CI** |
| --- | --- | --- | --- | --- | --- |
|  | **Test 1** | **Test 2** |  |  |  |
| Original PSQI | 4.05±1.90 | 3.92±1.75 | 0.40** | 0.62 | 0.49-0.72 |
| Malay validated PSQI | 2.82±1.55 | 2.74±1.35 | 0.42** | 0.61 | 0.48-0.71 |
| Components of the Malay validated PSQI:   1. Subjective sleep quality | 0.91±0.59 | 0.95±0.54 | 0.17* | 0.29 | 0.04-0.48 |
| 1. Sleep latency | 0.92±0.93 | 0.80±0.90 | 0.38** | 0.56 | 0.41-0.68 |
| 1. Sleep disturbances | 1.00±0.50 | 0.99±0.43 | 0.38** | 0.55 | 0.40-0.67 |

Note: n = 169
